# Symptomatic care of late-onset Alexander disease presenting with area postrema-like syndrome with prednisolone; a case report

**DOI:** 10.1186/s12887-022-03468-y

**Published:** 2022-07-13

**Authors:** Safoura Zardadi, Ehsan Razmara, Maryam Rasoulinezhad, Meisam Babaei, Mohammad Reza Ashrafi, Neda Pak, Masoud Garshasbi, Ali Reza Tavasoli

**Affiliations:** 1 Department of Biology, School of Basic Sciences, Science and Research Branch, Islamic Azad University, Tehran, Iran; 2 Present affiliation: Australian Regenerative Medicine Institute, Monash University, Clayton, VIC 3800 Australia; 3 Department of Medical Genetics, Faculty of Medical Sciences, Tarbiat Modares University, Tehran, Iran; 4 Myelin Disorders Clinic, Pediatric Neurology Division, Children’s Medical Center, Pediatrics Center of Excellence, Tehran University of Medical Sciences, Tehran, Iran; 5 Department of Pediatrics, North Khorasan University of Medical Sciences, Bojnurd, Iran; 6 Pediatric Radiology Division, Children’s Medical Center, Pediatrics Center of Excellence, Tehran University of Medical Sciences, Tehran, Iran

**Keywords:** Alexander disease, AxD type II, GFAP, Vomiting, Steroid

## Abstract

**Background:**

Alexander disease (AxD) is classified into AxD type I (infantile) and AxD type II (juvenile and adult form). We aimed to determine the potential genetic cause(s) contributing to the AxD type II manifestations in a 9-year-old male who presented area postrema-like syndrome and his vomiting and weight loss improved after taking prednisolone.

**Case presentation:**

A normal cognitive 9-year-old boy with persistent nausea, vomiting, and a significant weight loss at the age of 6 years was noticed. He also experienced an episode of status epilepticus with generalized atonic seizures. He showed non-febrile infrequent multifocal motor seizures at the age of 40 days which were treated with phenobarbital. He exhibited normal physical growth and neurologic developmental milestones by the age of six. Occasionally vomiting unrelated to feeding was reported. Upon examination at 9 years, a weak gag reflex, prominent drooling, exaggerated knee-deep tendon reflexes (3+), and nasal tone speech was detected. All gastroenterological, biochemical, and metabolic assessments were normal. Brain magnetic resonance imaging (MRI) revealed bifrontal confluent deep and periventricular white matter signal changes, fine symmetric frontal white matter and bilateral caudate nucleus involvements with garland changes, and a hyperintense tumefactive-like lesion in the brain stem around the floor of the fourth ventricle and area postrema with contrast uptake in post-contrast T1-W images. Latter MRI at the age of 8 years showed enlarged area postrema lesion and bilateral middle cerebellar peduncles and dentate nuclei involvements. Due to clinical and genetic heterogeneities, whole-exome sequencing was performed and the candidate variant was confirmed by Sanger sequencing. A *de novo* heterozygous mutation, NM_001242376.1:c.262 C > T;R88C in exon 1 of the *GFAP* (OMIM: 137,780) was verified. Because of persistent vomiting and weight loss of 6.0 kg, prednisolone was prescribed which brought about ceasing vomiting and led to weight gaining of 3.0 kg over the next 3 months after treatment. Occasional attempts to discontinue prednisolone had been resulting in the reappearance of vomiting.

**Conclusions:**

This study broadens the spectrum of symptomatic treatment in leukodystrophies and also shows that R88C mutation may lead to a broad range of phenotypes in AxD type II patients.

## Background

Alexander disease (AxD, OMIM: 203,450) is a rare, progressive autosomal dominant neurological disorder. According to a classification system proposed by Prust et al. AxD is categorized into two major groups: AxD type I (infantile subgroup) and AxD type II (juvenile and adult type) [1]. This system mainly focuses on clinical manifestations and distribution of brain magnetic resonance imaging (MRI) lesions. Infantile AxD is the most common type involving 63% of reported cases; this type is mainly characterized by profound intellectual disability and progressive neurologic deterioration, seizures, spasticity, megalencephaly, and white matter changes especially in the frontal area of the brain during the first 2 years of life [2, 3]. Intractable vomiting and tumor-like lesions have also been reported in the infantile subgroup [1]. The juvenile and adult form of AxD mainly affects patients with a slower clinical course. Bulbar signs, ataxia, palatal myoclonus, spastic paraparesis with hyperreflexia, and autonomic dysfunction are the most observed clinical manifestations. Motor and cognitive functions are interestingly more preserved in this group [1].

The area postrema syndrome consists of uncontrollable vomiting, brain stem lesions, and weight loss symptoms and has been reported in association with inherited white matter disorders such as AxD [4]. Mitochondrial leukodystrophies and hypomyelination with brain stem and spinal cord involvement and leg spasticity (HBSL; OMIM: 615,281) can also show area postrema-like syndrome [5].

Steroid therapy may be important and occasionally life-saving in adrenal insufficiency with X-adrenoleukodystrophy (X-ALD). In addition, symptomatic treatment of spasticity in HBSL (caused by *DARS* mutation) has been reported [6]. Here, we report clinical improvement of an AxD type II patient who presented area posterma-like symptoms. This study is a novel report on the symptomatic treatment of leukodystrophy and also is the first case report of AxD type II with R88C mutation in the Iranian population [2, 7–9].

## Case presentation

### Clinical manifestations

The patient was a 9-year-old boy who was initially reffered at the age of 6 years to our neurology department. He had been admitted to the gastroenterology ward due to persistent nausea and uncontrollable vomiting. He also showed a significant weight loss of about 6 kg over the previous 2 months. All gastroenterological assessments including abdominal ultrasound, barium swallow test, and upper gastrointestinal endoscopy were normal.

He showed non-febrile infrequent multifocal motor seizures with fixed upward gaze and head movements especially during breastfeeding at the age of 40 days. Because of frequent and abnormal scattered epileptiform discharges on electroencephalogram (EEG), phenobarbital was prescribed and led him being seizure-free. The drug was discontinued at 16 months of age and the patient had normal physical growth and neurologic developmental milestones by the age of six. He had been showing occasional vomiting, i.e., once or twice a month, unrelated to feeding without any remarkable etiology. To control this condition, proton pump inhibitors were prescribed and it gave rise to a relative improvement.

He was born at 38 weeks of gestational age through an uneventful normal cesarean section in a nonconsanguineous family. His birth weight and head circumference (HC) were recorded 3,050 g (z score = 0) and 37.0 cm (z score = 2), respectively. Over the first 2 weeks of his life, he was admitted due to prolonged physiologic jaundice with indirect bilirubin of 14 mg/dL which was resolved with phototherapy. While HC of his father and uncle was above the 2 standard deviations (SD) of their age, they had no clinical symptoms.

At the age of 6 years, the patient showed an episode of status epilepticus with generalized atonic seizures followed by severe occasional projectile vomiting and recurrent daily nausea in the early morning and sometimes at midnight. His seizures were controlled by taking sodium valproate and levetiracetam. He also showed significant weight loss of 6.0 kg (i.e., from 19.0 to 13.0 kg) over the following 2 months. At the first visit at this age, the patient had normal developmental milestones including speech, motor, and cognition. Upon examination, his weight and HC were 13.0 kg (z score = − 1) and 52.5 cm (z score = 2).

Ophthalmic examinations revealed a normal gaze in all directions and normal pupils’ reaction to light. Palatal myoclonus was not detected, however, nasal tone speech was obvious. He had a weak gag reflex and prominent drooling. Examination of other cranial nerves was normal. Knee deep tendon reflexes (DTRs) were exaggerated (3+), but others were normal (2+). Plantar reflexes were bilaterally downward. His gross motor function was valued as 1 out of 5 (1/5) based on the Gross Motor Function Classification System (GMFCS) scale.

All basic biochemical and metabolic tests such as thyroid and liver function tests, serum ammonia and lactate, urine organic acids profile, metabolic screen test (MS/MS), acylcarnitine profile, and serum amino acids chromatography (HPLC) were normal. The wrist X-Ray was consistent with the patient’s real age.

**Fig. 1:**
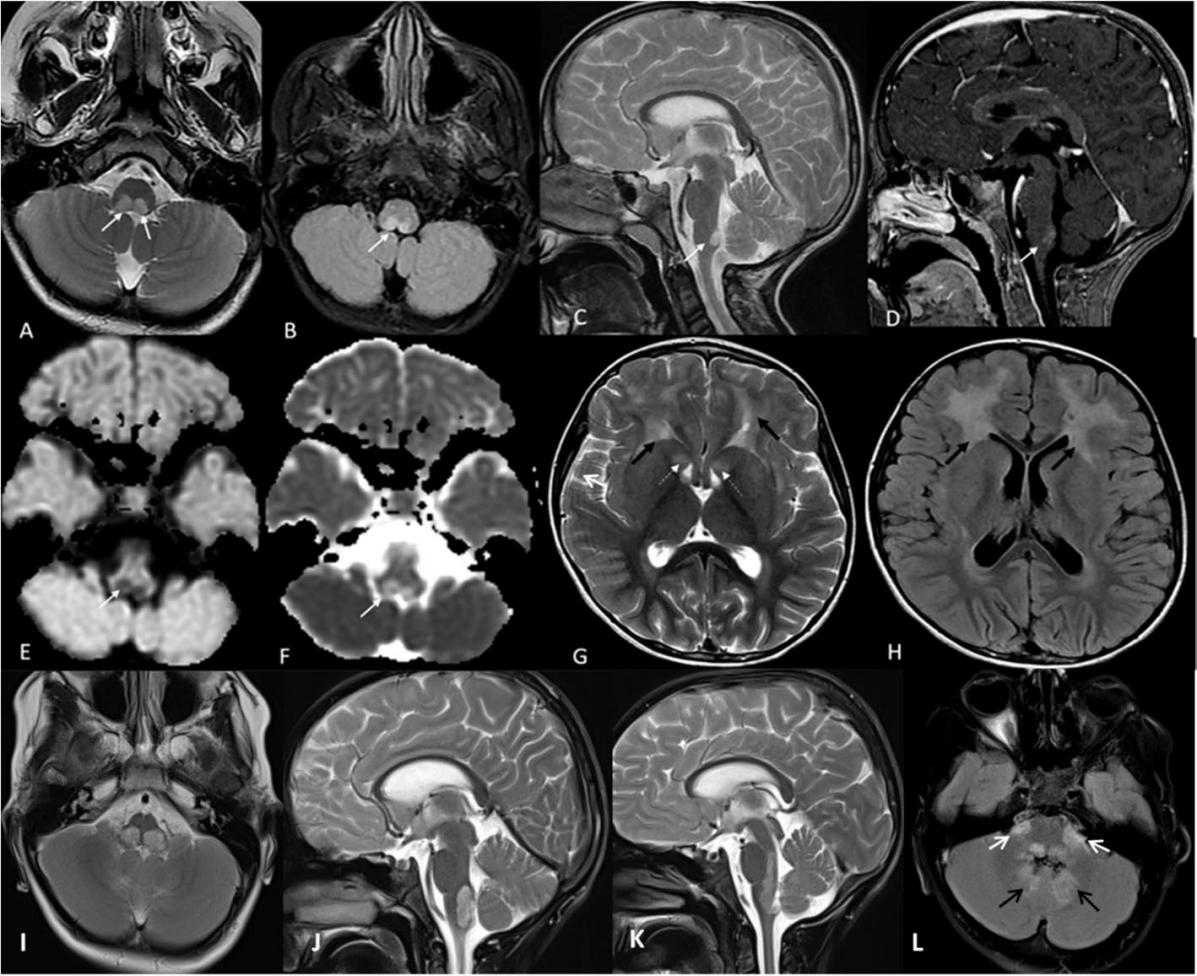
The patient’s brain magnetic resonance imaging (MRI) findings. MRI data demonstrated bilateral lesions (white arrows) in the posterior aspect of medulla oblongata near the floor of the fourth ventricle ( known as area postrema), which was hyperintense on T2-W (**A** and **C**) and FLAIR (**B**) sequences with some enhancements on T1-W post gadolinium administration sequence (**D**) and with no restricted diffusion on DWI (**E**) and ADC map (**F**) sequences. The involvement of periventricular white matter (especially in frontal lobes) with extension to subcortical white matter containing few small cysts (black arrows in **G** and **H**) and hyperintensity of basal ganglia in caudate heads (white dashed arrows in **G**). Third-row images belong to follow-up MRI at the age of 8 years including axial and sagittal T2-W (**I, J, K**) and Axial FLAIR (**L**); enlargement of the lesion in area postrema (**I** and **J**) extending to posterior pons (**K**) and involvement of bilateral middle cerebellar peduncles and dentate nuclei (white and black arrows in **L**, respectively)

The first MRI at the age of 6 years revealed faint bifrontal confluent deep and periventricular white matter signal changes, bilateral caudate nucleus involvement, significantly high in T2-W and low in T1-W images with garland and cystic changes around the tip of the frontal horns of the lateral ventricles, especially the left side (Fig. 1G and H). In addition, a hyperintensity tumefactive lesion was identified in the brain stem area around the floor of the fourth ventricle and area postrema (Fig. 1A–C). No restricted diffusion on diffusion-weighted imaging (DWI) (Fig. 1E) and apparent diffusion coefficient map (Fig. 1F) sequences were detected. Post-contrast T1-W images showed contrast enhancement in brain stem lesions (Fig. 1D).

According to the frontal predominance of symmetric white matter involvement, bilateral basal ganglia, and brain stem involvement, and post-contrast T1-W enhancement, AxD was highly suspicious. Interestingly, molecular study confirmed AxD diagnosis. The patient was prescribed to take oral prednisolone, 5–7.5 mg per day, which resulted in ceasing uncontrollable vomiting and weight gaining of 3.0 kg from 13.0 to 16.0 kg over the next 3 months of diagnosis. Speech therapy was also used to improve swallowing ability. The patient’s anti-seizure medications were continued.

At the age of 6.5 years, he showed sudden onset of episodic sympathetic symptoms, e.g., sweating, hyperpnea, and immediately oral secretion aspiration that gave rise to apnea and cyanosis. Each episode was lasting between 1 and 4 min, especially during speeching, and was irrelevant to feeding and consciousness. This was often exacerbated by fatigue. His EEG was also normal. Over time, vomiting and nausea disappeared but they relapsed on occasional attempts to discontinue prednisolone. Therefore, the steroid was continued at 5.0 mg/day and he was monitored for long-term steroid consequences. Drooling was continued intermittently but significantly improved after steroid therapy. He could tolerate eating small amounts of solid and liquid foods. Sodium valproate and levetiracetam were discontinued after 1 year of seizure-free at age of 7.5 years. Another EEG at age of 8 years revealed normal background with some scattered epileptiform discharges. On the last physical examination at 9 years of age, his HC and weight were 56.0 cm (z score = + 3) and 19.0 kg (z score= − 2), respectively. The repeated brain MRI at age of 8 years showed enlargement of the lesion in the area postrema (Fig. 1I and J) extending to posterior pons (Fig. 1K) and involvement of bilateral middle cerebellar peduncles and dentate nuclei (Fig. 1L).

### Molecular findings

Genomic DNA was extracted from the patient’s blood sample and subjected to whole-exome sequencing (WES) that was performed using the Illumina Hiseq4000 platform (Illumina, Inc., San Diego, CA, USA) to obtain an average coverage depth of ~ 100×. Data analysis, base calling, annotation of variants, and filtering were undertaken according to previous studies [10]. Segregation analysis was performed after doing Sanger sequencing. In essence, WES detected a heterozygous mutation, c.262 C > T; R88C in the exon 1 of the *Glial Fibrillary Acidic Protein (GFAP)* which was previously indicated to be pathogenic in infantile-onset AxD [11, 12]. Sanger sequencing confirmed that this mutation is *de novo*, i.e., neither of the parents carry the mutation.

## Discussion and conclusions

As identified by Van der Knaap et al. five typical MRI findings are often associated with type I AxD including (i) extensive cerebral white matter changes with frontal predominance, (ii) a periventricular rim with high signal on T1-W and low signal on T2-W images, (iii) abnormalities of basal ganglia and thalami, (iv) brain stem abnormalities, and (v) contrast enhancement of particular gray and white matter structures [13]. At least, 4 out of these criteria have to be considered to diagnose type I AxD. As atypical MRI features, the predominance of posterior fossa white matter abnormalities, brainstem, cerebellum, and spinal cord atrophy has also been detected in type II AxD [13]. According to Prust et al. classification system, the patient’s initial MRI features, and the age of clinical manifestations, the proband was suspected to be AxD type II; this was confirmed using WES and Sanger sequencing by identification of a pathogenic *de novo* missense mutation, (c.262 C > T; R88C) in the *GFAP*.

AxD is caused only by *GFAP* mutations that are present in about 95% of AxD patients. Most AxD cases are genetically imputed to *de novo* mutations, although familial cases have also been expected [8]. GFAP is a major intermediate filament protein in astrocytes [2, 14, 15]. More than 200 *GFAP* mutations have been identified in association with AxD mainly including missense and short in-frame indels [3]. R88C and R88S are the most prevalent *GFAP* mutations [1]. For example, it has been identified that of 22 Chinese children with AxD, 23.81% had R88C/R88S mutations [8]. R88C mutation also causes late-onset AxD; for instance, Gorospe et al. reported 2 cases of juvenile AxD with R88C mutation one patient manifested bulbar signs and cognitive defects, whereas another case was asymptomatic [8, 14, 16, 17]. The genotype-phenotype correlation has not been clearly looked into in cases with R88C/R88S mutations [1]. According to literature reviews, we suggest that R88C/R88S mutations may result in variable expressivity in AxD patients.

Macrocephaly is noticed in most patients with infantile AxD and some cases of late-onset disease [1]. The patient we discussed also showed macrocephaly since his infancy. In addition, a seizure is more evident in type I AxD than in type II [8]. The proband manifested in his seizure around his late neonatal period. Cognition is more preserved in late-onset AxD. Furthermore, our patient did not show spasticity and ataxia. These symptoms are observed in about half of cases with later-onset AxD [8, 18]. Autonomic dysfunction is an AxD late symptom with an average onset of 30 years [1], but the proband revealed episodes of autonomic dysfunction so soon, i.e., at the age of 6 years.

Different pathomechanisms may contribute to AxD pathology, e.g., immune system activation, cellular proteasome and autophagic response dysfunctions, and impaired excitatory neurotransmitters transport [19]. The partial effectiveness of a few symptomatic treatment methods such as lithium and ceftriaxone therapy has been demonstrated in AxD cases and animal models [3, 20], however, a few studies are available about this kind of treatment in AxD cases using anti-inflammatory drugs [3]. For example, a case report on a 3-month-old girl with AxD showed the short-term therapeutic effectiveness of steroids in the symptomatic treatment of intractable epilepsy [20]. To the best of our knowledge, there is a snippet of information on symptomatic treatment of vomiting and weight loss in AxD type II using steroids.

There is still a lack of knowledge about the symptomatic treatment of leukodystrophies. Here, we introduced a case with novel manifestations who responded to symptomatic treatment using steroids.

## Data Availability

The datasets used and/or analyzed during the current study are available from the corresponding author on reasonable request.

## Abbreviations

AxD: Alexander disease
DTRs: Deep tendon reflexes
EEG: Electroencephalogram
GFAP: Glial Fibrillary Acidic Protein
GMFCS: Gross Motor Function Classification System
HBSL: Hypomyelination with brain stem and spinal cord involvement and leg spasticity
HC: Head circumference
MRI: Magnetic resonance imaging
NSAIDs: Non-steroid anti-inflammatory drugs
